# Nutritional Value and Physicochemical Characteristics of Alternative Protein for Meat and Dairy—A Review

**DOI:** 10.3390/foods11213326

**Published:** 2022-10-23

**Authors:** Yan Zeng, Enhui Chen, Xuewen Zhang, Demao Li, Qinhong Wang, Yuanxia Sun

**Affiliations:** 1 National Engineering Laboratory for Industrial Enzymes, Tianjin Institute of Industrial Biotechnology, Chinese Academy of Sciences, Xiqidao No. 32, Airport Economic Area, Tianjin 300308, China; 2 National Technology Innovation Center of Synthetic Biology, Tianjin 300308, China

**Keywords:** alternative proteins, nutritional value, physicochemical characteristics

## Abstract

In order to alleviate the pressure on environmental resources faced by meat and dairy production and to satisfy the increasing demands of consumers for food safety and health, alternative proteins have drawn considerable attention in the food industry. However, despite the successive reports of alternative protein food, the processing and application foundation of alternative proteins for meat and dairy is still weak. This paper summarizes the nutritional composition and physicochemical characteristics of meat and dairy alternative proteins from four sources: plant proteins, fungal proteins, algal proteins and insect proteins. The difference between these alternative proteins to animal proteins, the effects of their structural features and environmental conditions on their properties, as well as the corresponding mechanism are compared and discussed. Though fungal proteins, algal proteins and insect proteins have shown some advantages over traditional plant proteins, such as the comparable protein content of insect proteins to meat, the better digestibility of fungal proteins and the better foaming properties of algal proteins, there is still a big gap between alternative proteins and meat and dairy proteins. In addition to needing to provide amino acid composition and digestibility similar to animal proteins, alternative proteins also face challenges such as maintaining good solubility and emulsion properties. Their nutritional and physicochemical properties still need thorough investigation, and for commercial application, it is important to develop and optimize industrial technology in alternative protein separation and modification.

## 1. Introduction

Meat and dairy products are important food items for humans. With the increase in the global population and the improvement of living standards, the consumption of meat and dairy products continues to grow. The global consumption of meat reached 355.5 million tons in 2021, up 4.5% from 2020 [1], and the demand for meat is expected to reach 455 million tons in 2050, with an increase of 76% compared to 2005 [2]. Meanwhile, the global milk production reached nearly 928 million tons in 2021, up 1.3% compared to 2020 [3] and 117.70% compared to 2010 [4].

Livestock production, which accounts for around 70 percent of all agricultural land and 30 percent of the land surface [5], brings greenhouse gas emission, deforestation, water pollution and land degradation, putting great pressure on the ecological environment [6]. The excessive intake of red and processed meat can increase the risk of obesity, cardiovascular disease, stroke and other diseases [7]. Conventional animal husbandry may excessively use hormones and antibiotics, leading to zoonotic virus transmission, parasites, pathogenic bacteria infection and other problems [8]. Additionally, ethical concerns about animal welfare and slaughter have driven many consumers to reduce or exclude animal proteins from their diets [9]. Thereby, there is a broad consensus in the food industry to seek alternative proteins for meat and dairy products to meet the requirement of consumers for safety and health, as well as animal welfare considerations and to ensure the sustainable development of the ecological environment.

Plant proteins are the most common animal alternative proteins. Commercial plant proteins include legume proteins such as soybeans, peas, chickpeas, feather fan beans, cereal proteins such as wheat, rice and oats, and nut proteins such as almonds, cashew nuts and walnuts. For example, the brands of Beyond Meat and Impossible Food in the United States have launched a series of plant meat products using pea protein and soybean protein as the main raw materials, respectively, while the Swedish company Oatly, listed in NASDAQ in 2021, has launched milk-free oat drinks as the core product [10].

In recent years, fungus proteins and algal proteins have received considerable attention in the field of alternative proteins, owing to their low dependence on land and climate, low resource consumption and high production efficiency. For example, the US company Nature’s Fynd, which employs *Fusarium flavolapis* from geothermal springs in Yellowstone Park to produce protein foods, raised more than USD 350 million in 2021 [11]. The Singapore company Sophie’s Bionutrients has developed dairy-free milk and cheese using chlorella protein powder as the main ingredient [12]. Meanwhile, although the large-scale industrial production has not been realized, insect proteins, which are closer to meat in terms of nutrition and taste, are also considered as potential substitutes for meat and dairy proteins [13].

The necessity and potential of animal alternative proteins have been well-recognized in the food industry. However, research on the nutritional value and techno-functional properties of animal alternative proteins is still at an early stage, and in this field, there are few reviews focusing on the comparison between plant, fungal, algal and insect proteins. Given that nutritional value and techno-functional properties are fundamental to the quality of protein-derived food, this review outlines the current knowledge of the nutritional value and techno-functional properties of alternative proteins from plants, fungi, algae and insects in order to support the research, product development and commercialization of alternative proteins.

## 2. Nutritional Value of Meat and Milk Alternative Proteins

Meat and dairy are important sources of protein, vitamin and mineral intake in the human diet. Considering that B vitamins and iron, which are rich in meat, and vitamin A, calcium and phosphorus, which are rich in milk, can be supplied by exogenous nutritional fortifiers added to animal alternative products, the protein content, amino acid composition and protein digestibility are the main criteria here for evaluating the nutritional value of meat and milk alternative proteins.

### 2.1. Protein Content

The protein content of meat is about 20%. For example, Lozano et al. measured the protein content of beef, chicken and pork as 19.6–21.6%, 22.5–23.4% and 19.9–23.1%, respectively [14]. Normal bovine milk contains about 3.5% protein [15]. For example, Bijl et al. reported that the protein content of skimmed milk was 3.30–3.69% [16]. Among plant proteins, the protein content of oilseeds is 40–65%, which is higher than that of beans at 18–36%, nuts at 15–30% and cereals (except wheat at 16%) at 8–11% [17,18,19,20,21,22,23,24,25,26,27]. As for pseudocereals, the protein content varies from 9.1 to 16.7% in quinoa, 13.1 to 21.5% in amaranth and 5.7 to 14.2% in buckwheat [28,29]. In addition, as shown in Table 1, *Moringa Oleifera* defatted leaves, which belong to the novel plant protein source leafy plant material, have a protein content of 24.1% [30]. The protein content of edible fungi is generally about 19–37% of dry weight [31,32], but the protein content of *Fusarium venenatum* reaches 45–54% [33] and can be further improved by optimizing the culture medium and fermentation conditions [34]. In algae, cyanobacteria has a higher protein content, such as spirulina, whose protein content can account for about 60–70% of dry weight [35], followed by green algae and red algae, such as chlorella, whose protein content can account for about 51–58% of dry weight [36,37]. Brown algae has the lowest protein content of about 10–15% [38]. The protein content of insects is 35–60% of dry weight and 10–25% of wet weight, which is close to the common animal protein content [39,40]. It is very important to note that the protein content of alternative proteins depends on the species sources and cultivation environments. Furthermore, the extraction and processing conditions have a great influence. For example, soybean protein concentrate with a protein content of 65–70% and soy protein isolate with a protein content of 85–90% can be prepared from defatted soybean flour with a protein content of 50–55% by acid leaching or alkali–soluble acid precipitation and alcohol–soluble acid precipitation, respectively [41].

Most alternative protein food products on the market today are derived from plant proteins, including plant protein meat and plant protein milk. Cutroneo et al. found that the protein content of plant-based hamburger meat patties and meatballs was lower than that of corresponding animal meat products [42]. Chalupa-Krebzdak et al. compared the protein content of commercially available milk and plant milk and found that in plant milk, the protein content of almond milk was the lowest at about 0.31–0.59 g/100 mL, and the protein content of soy milk was the highest; however, its protein content of 2.50–3.16 g/100 mL was still lower than the protein content of 3.15–3.37 g/100 mL in real milk [43]. In addition to plant proteins, other alternative protein-derived foods may also not provide animal-food equivalent proteins. For example, similar to Beyond Meat and Impossible Food plant-based burger patties, Quorn mycoprotein-based chicken analog nuggets had a lower protein content than commercially available real chicken nuggets.

The relatively low protein content in final alternative protein food products may be due to the difficulty in the industrial level production of alternative proteins with a high protein content. In addition, alternative proteins may not be able to be added to foods in large quantities due to their poor techno-functional performance in food processing. Therefore, in order to resolve the problem, it is necessary to develop novel technologies for alternative protein extraction with a high purity and low cost and to enhance the physicochemical properties of alternative protein through various modification methods.

### 2.2. Amino Acid Composition

Amino acids are the basic building blocks of proteins involved in the regulation of key metabolic pathways and processes that are crucial for the growth and maintenance of organisms. The nine essential amino acids, including lysine, histidine, isoleucine, leucine, methionine, phenylalanine, threonine, tryptophan and valine, cannot be synthesized by the human body and can only be supplemented by diet; thus, the content and coverage of these essential amino acids are very key indicators in the evaluation of protein quality.

Generally, alternative proteins have a lower content of essential amino acids compared to animal-based proteins. In the report of Gorrissen et al. [44], although the essential amino acid content of soy protein isolate, black rice protein isolate, pea protein isolate and corn protein isolate reached 27%, 28%, 30% and 32%, respectively, and met the essential amino acid content recommended by the WHO/FAO/UNU, they were still lower than the essential amino acid content of 39%, 43% and 34% in milk, whey and casein. Similar to the protein content, the variations in the essential amino acid content in different alternative proteins can be explained by genetic characteristics [21,24,28] as well as environmental conditions. For example, Präger reported that the essential amino acid content of the same quinoa cultivar ranged from 24% to 30% when harvested in different years [45]. The essential amino acid content of fungal protein is 25–45% [46]. For example, the essential amino acid content accounts for 31.59–38.54% in *Pleurotus geesteranus* [32]. As for algae, Mišurcová et al. reported that the essential amino acid contents of *Spirulina Pacifica* and *Spirulina platensis* were almost 20% [47]. Similarly, Dewi et al. found that the essential amino acid content of *Spirulina* sp. was 21.80% [35] and was significantly lower than the essential amino acid content of 42–48% in red and green algal proteins [48]. The essential amino acid levels in insect proteins are comparable with soybean proteins but lower than for casein [40,49].

Animal-derived proteins contain the full set of essential amino acids. The main amino acids of meat include glutamic acid, lysine and leucine. The content of glutamine, proline, leucine, lysine and valine together accounts for 50% of the total amino acids in milk and cheese. In contrast, Bessada et al. [50] reported that plant proteins lacked sulfur-containing amino acids and/or certain essential amino acids, especially lysine, tryptophan and threonine. Apart from the essential amino acid content, there are big differences in the amino acid composition between various alternative proteins. Soy proteins are relatively rich in lysine, arginine, leucine and aspartic acid but deficient in methionine, cystine and tryptophan, while cereal proteins have a relatively high content of leucine and methionine but a low content of lysine, threonine and tryptophan [51]. The predominant amino acids in pseudocereals are glutamic acid, arginine and aspartic acid, and the sulphur amino acids are found the least [52]. Algal proteins are rich in glutamic acid and aspartic acid and have a higher content of sulfur-containing amino acids than plant proteins but have a lower content of leucine, lysine and histidine [53]. Compared to plant and algal proteins, fungal proteins are rich in lysine and leucine, and their content of sulfur-containing amino acids (such as methionine and cysteine) is also higher [54]. As for most insect proteins, sulfur-containing amino acid is the limiting component [55].

Due to the amino acid unbalance, it is hard to completely replace animal proteins in food just by one alternative protein itself, even if an alternative protein material with a high protein content is obtained. Thus, in addition to supplying exogenous amino acid as fortifiers, it is a good choice to use an alternative protein mixture, such as plant proteins combined with fungal proteins to formulate a new or optimized product.

### 2.3. Digestibility

Proteins can only be assimilated by the human body after digestion into tripeptides, dipeptides and amino acids. Protein digestion begins with pepsin, but most occurs in the intestinal lumen under the action of pancreatic digestive enzymes such as trypsin, chymotrypsin, elastase and carboxypeptidase. Then, the peptidases-associated small intestinal epithelial brush border breaks down the peptides into tripeptides, dipeptides and amino acids, which are subsequently absorbed by the intestinal epithelium. The proteins, peptides and even free amino acids that have not been digested and absorbed in the small intestine eventually end up in the large intestine, where they will be fermented by the gut microbiota [56]. Therefore, the digestion and absorption behavior of proteins have an important influence on their actual nutritional value. The digestibility of proteins is closely related to their structural properties. A large molecular weight and complex structure as well as a high degree of folding and aggregation will lead to low protein digestibility [57]. For example, the digestibility of meat, milk and plant proteins is negatively correlated with the degree of β conformation (β sheet and β turn) in their secondary structure [58]. Furthermore, the residual cellulose, pectin, glucan and mannan in plant proteins, fungal proteins and algae proteins and the residual crustacean in insect proteins can hinder the contact between digestive enzymes and proteins, affecting digestibility. In addition, non-animal proteins may also have protease inhibitors and antinutritional factors such as lectins, phytic acid and tannin polyphenols, which may adversely affect the digestibility of products [59].

The protein digestibility corrected amino acid score (PDCAAS) and the digestible essential amino acid score (DIAAS), based on fecal digestibility and ileal digestibility, respectively, are internationally recommended to assess the bioavailability of amino acids. Given that the ileal digestibility excludes the interference of the metabolically transformed proteins of the large intestine microbes, the DIAAS score is more accurate than the PDCAAS score. Nevertheless, the lack of ileal digestion data of a large number of foods has been an obstacle in the development of the DIAAS score.

All animal sources of protein have PDCAAS values equal to or above 1, such as skimmed milk, milk protein isolate and casein with a PDCAAS score of 1 [60]. As shown in Table 2, in general, plant proteins have lower PDCAAS scores than meat and milk because of their lower digestibility and certain insufficient essential amino acids [61,62,63,64,65]; the exception are soy protein isolate [61] and potato protein [62] with PDCAAS scores of 0.92–1.00 and 1.00, which are close to some proteins from meat and dairy. Unprocessed legumes generally have a PDCAAS score of less than 0.8 but can be partially improved after processing. For example, the PDCAAS scores for peas and pea protein isolates are 0.64 and 0.82 [17], respectively. Nuts, cereals and pseudocereals, such as almonds [64,65], walnuts [66], oats [17], rice [67], wheat [67], quinoa [68], amaranth [69] and buckwheat [70] have lower PDCAAS scores than legumes: 0.39, 0.55, 0.64, 0.53, 0.54, 0.68, 0.64 and 0.54, respectively.

Edible mycoproteins have a PDCAAS of 0.35–0.70 [31], but the mycoprotein from *Fusarium venenatum* has a PDCAAS score of 0.996, which is superior to beef and chicken [33,71]. The PDCAAS score of yeast protein concentrate extracted using isoelectric precipitation also achieves 0.90 [71,72]. As for algae, the microalga *Chlorella* vulgaris has a PDCAAS score of 0.63 [36], the red alga *Palmaria palmata* has the highest PDCAAS score of 0.69 in vitro digestive system, while the brown alga *Fucus vesiculosus* has the lowest PDCAAS score of 0.08 [73]. The PDCAAS score of insects is close to that of legumes [74,75]. For example, the PDCAAS score of *Tenebrio molitor* protein obtained from freeze-drying, defatting, extrusion and formic acid hydrolysis is 0.79–0.82 [76]. The PDCAAS scores of crickets, moth caterpillars, grasshoppers and termites tested by Oibiokpa et al. were 0.73, 0.42, 0.46 and 0.42, respectively [77].

According to the FAO recommendation, the DIAAS score of high-quality protein and excellent quality protein should be higher than 75 and 100, respectively [78]. Meat (beef, pork, chicken, etc.) and milk proteins (casein, milk protein concentrate, skimmed milk powder, whey protein concentrate and whey protein isolate, etc.) have DIAAS scores above 100 [78,79,80]. Potato protein has the highest DIAAS score of over 100 in plant proteins [78]. Soy products such as soy flour and soybeans have a DIAAS of nearly 100, while pea protein concentrate has a DIAAS of only 73. Nuts and cereals such as almonds [65], wheat [51], rice [61] and oats [63] have relatively low DIAAS values of 40, 43, 60 and 66, respectively. There have been very few studies on the DIAAS score of fungal, algal and insect proteins. Ariëns et al. applied the INFOGEST in vitro digestion protocol to evaluate the DIAAS score of food and found that, although this method was not suitable for filamentous fungal proteins, the DIAAS score of yeast concentrate protein was measured to be 97.2 [71]. Although algae from the genera *Arthrospira*, *Fasciola*, *Chlorella*, *Dunaliella* and *Rhodococcus* have been used in food, according to Wang et al., there had been no published data on the DIAAS score of *spirulina* and its related proteins until 2020 [53]. Recently, Malla et al. studied the DIAAS scores of carp beetles, crickets and soldier flies, and the DIAAS scores of these insects were 64–92, among which the crickets had the highest DIAAS score [75].

Based on the PDCASS and DIAAS data, the soy protein isolate and potato protein from plant proteins, the mycoprotein of *Fusarium venenatum* and yeast protein from fungal proteins and crickets from insect proteins are good alternative protein candidates. The unsatisfactory digestibility of algal proteins may be related to the presence of rigid cell walls and the large amounts of phenolic compounds. Considering that polysaccharide hydrolases such as cellulase can enable the weakening or disruption of the cell wall structure of algae, thereby facilitating the release of bounded phenolic compounds, the combination of enzymatic and solvent extraction can be considered a good attempt to improve the recovery and digestibility of algal proteins and other alternative proteins.

**Table 2:** PDCAAS and DIAAS of some reported alternative proteins.

Protein Type	Protein Sources	PDCAAS	DIAAS	Reference
plant protein	soy protein isolate	0.92–1.00	90	[61]
	potato protein	1.00	>100	[62,78]
	pea	0.64	73	[17,63]
	lupin	0.40–0.80	65–72	[64,81]
	almond	0.39	40	[65]
	walnut	0.55	NA	[66]
	oat	0.64	66	[63]
	brown rice	0.59	60	[63]
	wheat	0.54	43	[51,67]
	quinoa	0.68	NA	[68]
	defatted *Moringa Oleifera* leaves	0.61	NA	[30]
fungal protein	mycoprotein (from *Fusarium venenatum*)	0.91–0.996	NA	[33,71]
	yeast protein concentrate	0.82–0.90	97	[71,72]
algal protein	*Chlorella vulgaris*	0.63	NA	[36]
	*Palmaria palmata*	0.69	NA	[73]
	*Fucus vesiculosus*	0.08	NA	[73]
insect protein	*Hermetia illucens* L.	0.75	73	[74]
	yellow mealworm (*Tenebrio molitor*)	0.79–0.82	64	[75]
	banded cricket (*Gryllodes sigillatus*)	0.65	92	[75]

NA: data not available.

## 3. Physicochemical Characteristics of Animal Alternative Proteins

Proteins are widely employed as ingredients in foods, not only for their high nutritional value but also because of their physicochemical characteristics, which play a key role in the texture and organoleptic properties of the final products. For example, the quality of minced meat products is closely related to the gelation, emulsion and water retention property of muscle proteins [82]. Solubility is important in milk beverages, emulsification is important in processed cheese, viscosity is important in fermented milk, and foaming is vital for ice cream [83]. In addition to the intramolecular and intermolecular interactions of proteins, due to the hydrophilic and hydrophobic groups, sulfhydryl groups and charged side chains in the structure, proteins also can interact with fats, carbohydrates and other components in food through hydrogen bonds, disulfide bonds, electrostatic attraction and hydrophobic interactions to form aggregates, clusters and three-dimensional networks [84]. It has been revealed that the sulphide cross-linking of myofibrillar protein that coated oil droplets in a protein matrix could stabilize and strengthen the protein–emulsion composite gels in comminuted muscle foods [82].

### 3.1. Solubility

The solubility of proteins refers to their ability to disperse in an aqueous environment. Many functional properties of proteins, such as gel forming, emulsifying and their foaming abilities are directly related to their solubility. Therefore, solubility is important for the potential applications of proteins [85]. The solubility of proteins depends on structural characteristics such as the amino acid sequence, surface charge, surface hydrophobicity and molecular size and external factors such as temperature, pH and ionic strength. Generally, proteins with a large surface area, high surface charge repulsion and loose molecular structure are easy to be wetted in an aqueous media and effectively dissolved [86]. For example, due to the presence of more hydrophilic amino acid residues such as lysine, histidine, aspartic acid and glutamic acid, the water solubility of lentil protein isolate and pea protein isolate is higher than that of chickpea protein isolate [87]. Apart from the composition of amino acids, the relative spatial distribution of hydrophobic and hydrophilic amino acids also has an important effect on solubility by affecting the protein surface wettability, water adsorption and solvation [86]. The solubility of proteins usually rises with temperature but rapidly decreases when denatured and inactivated at high temperatures. At an isoelectric pH, the net charge of protein molecules is zero, and the protein molecules easily collide with each other and aggregate and precipitate; thus, the solubility is the lowest. In addition, at a low ionic strength, ions can act as a barrier to prevent proteins from attracting aggregation, while at high ionic strengths, salts and proteins compete for water molecules, making the proteins less soluble.

The nitrogen soluble index (NSI) is the percentage of water-soluble nitrogen to the total nitrogen or the percentage of water-soluble protein to the total protein, which is usually used to evaluate the solubility of proteins. The NSI is 33% for pork [88], 37% for beef [89] and 98.6% for whey protein concentrate [90]. It was found that the NSI of soybean protein isolate was the highest under a neutral pH but was still below 40%. The NSI of pea protein concentrate and wheat protein isolate was lower than 20%, while the NSI of rice protein concentrate was close to 0 [91]. The NSI nitrogen soluble index of the rapeseed protein, soybean protein concentrate and wheat gluten protein tested were 19.5%, 42.9% and 10.3% [92], respectively. Samard et al. used soybean protein isolate, wheat gluten and corn starch to produce tissue protein using high moisture extrusion and found that the NSI of the raw materials was 24.15%, while the NSI of the textured protein product using extrusion at 50% moisture and 130 °C was only 7.4% [93]. These reports indicate that conventional plant proteins are generally less soluble than animal proteins, and their processed food also have lower solubility. However, the protein concentrate prepared from dried alfalfa (*Medicago sativa* L.) leaves using alkali solubilization–acid precipitation showed a moderate NSI of about 50% from pH 5.5 to 10 [94], and the protein concentrate isolated from sugar beet (*Beta vulgaris* L.) leaves using enzyme-assisted extraction showed a high NSI of 98.71% at pH 7.5 [95].

There are few reports in the literature on the solubility of fungal, algal and insect proteins. The protein F60 extracted from *Pleurotus nebrodensis* using the alkaline leaching staged salting-out method showed its highest NSI of 27.32% at pH 8.0 [96]. The NSI of the ectomycorrhizal fungi Amanita was up to 32% at pH 4.0, lower than 20% at pH 6.0 and 8.0 and rebounded to 27% at pH 10.0 [97]. The *Arthrospira* (*Spirulina*) *platensis* protein isolate presented a U-shaped solubility curve with the minimum NSI of 6.2% at pH 3 and the maximum NSI of 59.6% at pH 10 [98]. Zielińska found that protein from *Tenebrio molitor*, *Gryllodes sigillatus* and *Schistocerca gregaria* showed the lowest NSI of only 3%, 4% and 8% at around pH 5, respectively. However, their NSI significantly increased with pH and reached the maximum of 97%, 96% and 90% at pH 11.0, respectively [99].

It is speculated that the NSI of alternative proteins significantly increases under strong alkaline conditions, not only because the proteins are far from the isoelectric point but also because the residual cell walls or chitin exoskeletons of the protein raw material are destroyed and degraded under strong alkaline conditions, promoting protein solubility. Thus, the conventional method of alkali solubilization and acid precipitation, which utilizes the difference in the protein solubility in acids and alkali, is suggested in the extraction of alternative proteins. In addition, alkali treatment also can be combined with other strategies to modify alternative proteins to improve their solubility and the related techno-functional properties (Table 3) [100,101].

### 3.2. Gelation Properties

The gelation of proteins refers to the process of depolymerization, extension, orderly aggregation and cross-linking of peptide chains to form a continuous network structure after protein denaturation. Protein gel can adhere to particles, fix fat and retain water to improve emulsion, foam stability and increase the consistency of food. Gelation properties have an important effect on the structure and taste of semisolid foods such as meat and cheese. There have been a large number of studies on the gel formation mechanism and the influencing factors of animal protein. However, except for soybean protein and pea protein, there are few systematic studies on the gel properties of other animal alternative proteins, and it is even difficult to find articles dealing with the gelation of microbial proteins in 2021 [102].

The gelation property of proteins can be evaluated by: the minimum gelling concentration; rheological parameters such as the storage modulus and loss modulus; and texture analysis parameters such as hardness and elasticity. Generally, protein gels with a high cross-linking density have a higher storage modulus and loss modulus, the storage modulus is significantly higher than the loss modulus [103], and the hardness and elasticity of the protein will both increase with its gel stability. For example, soy protein isolate, pea protein isolate and lupin protein isolate have the minimum gelling concentration of 10%, 12.5% and 14% at pH 5.5 and 90 °C, respectively. At the same concentration, the storage modulus, loss modulus and hardness of soybean protein isolate, pea protein isolate and lupin protein isolate sequentially decrease, indicating that the gel performance of the soybean protein isolate is better than that of the pea protein isolate and lupin protein isolate. The poor gel performance of the lupin protein isolate may be a result of its large amount of disulfide bonds which induce a stable structure that is difficult to denature and dissociate under heating. Meanwhile, not enough available free sulfhydryl groups indicates a weak intermolecular cross-linking in the protein [104].

After heating in boiling water for 1 h, the minimum gelling concentration of the *Pleurotus ostreatus* protein concentrate was 2% and was higher than the value of 6% of *P. tuber-regium sclerotia* [105]. Under the same conditions, the minimum gelling concentration of *Termitomyces umkowaan* and *Auricularia auricula* reached 10% and 18% [106]. Nevertheless, given that only the minimum gelation concentration has been employed to assess the gel properties, and the reported fungal proteins are too few, the gelation conditions are harsh, and more data are needed to support the gel performance of fungal proteins. Chronakis et al. reported that 1.5% of spirulina protein isolate could form a gel at 90 °C [107], the protein-rich *Chlorella.* extract at the concentration of 9.9% (*m*/*v*) could form a weak gel at 61 °C;

However, the pH adjustment or addition of NaCl reduced its elasticity and hardness. It was speculated that the electrostatic interaction between proteins was the main driving force for the formation of the *Chlorella* extract gel [108]. At the concentration of 30% *w*/*v* and at pH 7 or 10, the dispersed protein fractions of mealworms, superworms, lesser mealworms, house crickets and Dubia cockroaches could form gel after heating at 86 °C for 10 min [109]. Honey bee raw powder, black cricket protein isolate and migratory locust protein concentrate showed the critical gelling concentrations as 5%, 6.5% and 20%, after heating at 85°C for 30 min, 90 °C for 15 min and 86 °C for 10 min, respectively [110,111].

The forces to form and maintain protein gels include hydrophobic interactions, noncovalent cross-links such as hydrogen bonds and electrostatic interactions and covalent cross-links such as disulfide bonds. Various physical, chemical and biological methods are used to construct and regulate the structure of protein gels. For example, the proton of glucono delta-lactone and the calcium ion of calcium sulfate can shield the surface negative charge repulsion of denatured soybean protein to promote protein aggregation for gel formation [112]. After trypsin hydrolysis, the content of acidic amino acids in oat protein decreases, the charge repulsion under neutral or alkaline conditions simultaneously decreases, and the hydrophobic effect is enhanced, resulting in the oat protein hydrolysate having a gel strength comparable to that of soy protein and egg white protein at a neutral pH and pH 9.0 [113]. As the protein was unfolded and disulfide cross-linking was enhanced in the acylation modification and guar gum conjugation, the minimum gelling concentration of pea protein concentrate decreases to 7%. [114]. At a neutral pH, 12% of native pea protein concentrate cannot form a gel under 90 °C but shows good gelling properties at 70–90 °C after atmospheric pressure cold plasma treatment. In addition to promoting protein unfolding and protein fibril aggregate formation, atmospheric pressure cold plasma treatment increases the protein surface hydrophobicity and exposes free sulfhydryl groups, facilitating the formation of hydrophobic interactions and disulfide bonds and leads to gels with enhanced mechanical properties [115]. Similarly, high hydrostatic pressure can result in isolated cowpea protein gels without heating [116]. High-pressure homogenization can improve the gel stiffness of *Spirulina* protein concentrate [117]. In addition to novel technologies, new raw materials have also gained interest in the field of protein gel research. For example, Nieuwland et al. reported that duckweed gels were much stronger than soy and only slightly weaker compared to egg white protein at pH 7.0, indicating its great potential as an alternative protein [118].

### 3.3. Emulsion Properties

Proteins are hydrophilic and hydrophobic and can be dispersed and adsorbed around oil droplets to form a viscoelastic film layer to reduce the interfacial tension between the oil phase and the water phase and prevent oil droplets from coalescing to achieve the emulsification effect. A good protein emulsifier should meet the requirements of rapid dispersion at the oil–water interface, sufficient structural and conformation unfolding, a complete interfacial film and good viscoelasticity.

Emulsifying capability (EC), emulsifying stability (ES), the emulsifying activity index (EAI), the emulsifying stability index (ESI) and droplet size distribution are widely used to evaluate the emulsifying performance of proteins. The emulsifying properties of plant proteins from soy, peas, lentils, broad beans, chickpeas, rapeseed and flaxseed have been extensively studied [119,120]. Soy protein isolate extracted using isoelectric point precipitation has a higher ESI, EC and EAI than that extracted using salting-out extraction. Soybean protein isolate, pea protein isolate, chickpea protein isolate and lentil protein isolate extracted using isoelectric point precipitation had similar EC, EAI and ESI values, and their mean droplet sizes showed no difference, suggesting their emulsifying properties were similar [121]. Zhao et al. found that the emulsification properties of commercially available legume protein isolates at pH 7.0 were ranked from high to low as soybean protein isolate, pea protein concentrate, wheat protein isolate and rice protein concentrate. The EAI of soybean protein isolate was higher than 100 m^2^/g, and its ESI was longer than 15 min, while the EAI of the rice protein concentrate was less than 10 m^2^/g, and its ESI could not be detected [91]. Similar to their solubility and gel properties, the emulsifying properties of proteins are not only related to their structural features but are also affected by many environmental conditions including oil phase volume fraction. Burger found that pea protein isolate had a better emulsification performance than soybean protein isolate in the system with an oil content less than 10%. However, if the oil content increased, the interface film formed by the pea protein isolate became unstable due to its limited molecular flexibility, the dispersed droplets underwent collision polymerization and induced larger particle sizes and a worse emulsification performance compared to soybean protein isolate [122].

The *Pleurotus tuoliensis* protein extracted using alkali-soluble acid precipitation could be rapidly adsorbed to the oil–water interface at 0.1 mg/mL after pH-cycle treatment, and the emulsion system formed with an oil content of 10–50% was stable for 30 days without delamination [123]. Similarly, the *Phlebopus portentosus* protein isolate had the ability to form emulsions [101]. The EAI and ESI of the protein from different *Pleurotus ostreatus* tested were 1.55–10.28 m^2^/g and 3.64–180.27 min, respectively [105]. The oil–water mixture of 1.0% chlorella protein or 3.7% *Phaeodactylum tricornutum* protein with an oil content of 5% could form a highly stable emulsion under high-pressure homogenization. The average particle size of the emulsion droplets varied very little throughout 7 days of storage [124]. Protein isolated from *Chlorella pyrenoidosa* showed the highest EAI value of 63.58 m^2^/g at pH 3.0, while protein isolated from *Arthospira platensis* showed the lowest EAI value of 30.83 m^2^/g at pH 5.0. The ESI values of the proteins isolated from *Chlorella pyrenoidosa*, *Arthospira platensis* and *Nannochloropsis oceanica* were highest at pH 5.0 at 15–18 min [125]. Cricket protein hydrolysate prepared using alcalase had the EAI of 7–32 m^2^/g [126]. Water-soluble proteins extracted from two species of grasshoppers, *Patanga succincta* and *Chondracris roseapbrunner*, had an EAI of 29.23 and 36.96 m^2^/g and an ESI of 15.67 and 33.34 min, respectively [127]. Jiang et al. found that salting-assisted extraction had an influence on the emulsification performance of protein isolate from *Tenebrio molitor* larvae; the EAI of the protein increased from 21.99 m^2^/g by adding sodium chloride during the alkaline extraction to 55.50 m^2^/g by adding ammonium sulfate during the acid precipitation [128].

At present, the emulsification performance of most alternative proteins is far from that of animal protein emulsifiers such as whey protein isolate, ovalbumin and casein. Physical, chemical and biological methods can be used to modify protein structures [114], and the preparation process of emulsion systems also can be optimized to ameliorate the emulsifying properties of alternative proteins [129]. For example, extruded soy protein concentrate had a lower α-helix content and higher β-sheet content compared to the native protein so that its flexibility increases during homogenization, and its EAI and ESI subsequently increased [130]. After succinylation, the negative charge on the surface of oat protein increased, and electrostatic repulsion occured, and the protein dimer dissociated, inducing an improved emulsifying property [131]. After being modified by ultra-high-pressure composite glycation or modified by glutaminase deamidation and succinylation, *Spirulina phycocyanin* showed good hydrophilic and lipophilic properties to disperse quickly in the emulsification interface, and its EAI and ESI were significantly improved [132,133].

### 3.4. Foaming Properties

Foam imparts body, smoothness and lightness to food and is important to several categories of foods, including meringues, whipped toppings and leavened bakery products. After rapid whipping, protein diffuses and adsorbs at the air–liquid interface, then unfolds at the interface with the orientation of the hydrophilic groups to the liquid phase and the hydrophobic groups to the gas phase. The anchored protein with a conformational rearrangement aggregates using intermolecular interactions to form continuous films that trap and encapsulate bubbles dispersed in the liquid phase to form foams [134]. Good hydrophobicity, viscosity and dispersion properties are helpful to the foaming ability of proteins, and a reduction in the surface charge is beneficial to the maintenance of protein foam stability [135].

Foaming capacity (FC), which can be expressed by calculating the increased volume percentage in the foaming of the dispersion, and foam stability (FS), which can be expressed by the percentage of foam remaining after the dispersion is left for a specific time, are widely used to evaluate the foaming properties of proteins. For example, the FC and FS of commercially available egg white protein, soy protein isolate and skimmed milk powder are 202%, 130%, 92% and 81%, 82%, 84%, respectively, indicating that egg white protein has the best foaming ability, followed by soybean protein and skimmed milk powder [136]. Lafarga et al. reported that protein isolates from lentils, cowpeas, broad beans, soybeans, peas, kidney beans, chickpeas, cauliflower and red beans, using alkaline solubilization extraction, all had higher foaming properties and foam stability at pH 2.0 and pH 10.0 than at other pHs; therein, the faba bean protein isolate had the best foaming performance, with an FC of 56.7% both at pH 2.0 and pH 10.0 [137]. The foaming properties of alternative proteins are not only dependent on structural factors such as the amino acid composition and molecular size, shape and conformational flexibility but are also affected by environmental pH, protein concentration and solubility. The FC of vicilin in green pea protein is better than that of legumin, whereas a completely opposite result is observed in FS. It is speculated that the better solubility, the less rigid conformation structure and the small molecular weight endow vicilin absorbed rapidly at the air–water surface, thus contributing to its better FC, whereas legumin containing higher α-helix content has a good ability to form a thick and cohesive film that can effectively prevent the collapse of air bubbles for a longer time [138]. Similarly, commercial rice endosperm protein hydrolysate has a comparable foaming performance (FC: 128%, FS: 83.6%) to skimmed milk powder (FC: 117%, FS: 89.9%) and whey protein isolate (FC: 127%, FS: 81.5%), which may be ascribed to the smaller size, higher solubility, flexibility and exposed hydrophobicity of the resulting hydrolysate compared to the intact counterpart. [139]. Yellow pea protein isolate extracted at pH 10.0 has a strong ability to reduce the air–water surface tension with a higher FC value [140]. Conversely, the flaxseed protein isolate and protein concentrate show good FC and FS at pH 3.5. This difference in foaming performance is likely due to the fact that in the yellow pea protein isolate, the increased net charges at an alkaline pH increase protein flexibility, while in flaxseed protein, the enhanced protein flexibility is contributed to the increased hydrophobic interactions at an acidic pH [141].

Algal proteins show good foaming properties, while insect proteins show moderate foaming properties. For example, protein extracted from *Chlorella pyrenoidosa* using a three-phase partitioning technique had an FC and an FS of 95% and 97%, respectively [142]. The FC and FS of pure *spirulina* phycocyanin were 158.2% and 118%, respectively [143], while the FC and FS of insect proteins from *Gryllodes sigillatus*, *Tenebrio molitor* and *Schistocerca gregaria* were 99.0%, 32.67%, 32.0% and 92.0%, 30.33%, 6.17%, respectively [103]. The foaming properties of the *spirulina* protein extracted using isoelectric precipitation were greatly affected by pH and positively correlated to protein solubility; its highest FC was over 250%, and its FS was 56.5%, respectively [98]. Similarly, the insect protein extracted from depigmented *Haematococcus pluvialis* using alkali-soluble acid precipitation had a better foaming performance at an alkaline or mildly acidic pH, with an FC and FS of 88.32% and 89.62% at pH 10.0 and 83.78% and 84.31% at pH 3.0, respectively [144]. Due to the greater unfolding degree and exposure of the aromatic groups, the FC of alkaline-extracted glutelin from the *Cordyceps militaris* fruit body was 151%, higher than the FC of the extracted albumin and globulin of 50% and 101.33%, respectively; however, its FS was lower due to its poor solubility and low viscosity [145]. The FC value of the protein concentrate from *Pleurotus ostreatus* was 109.1–144.5%, and the FS value was 36.3–47.5% [105]. However, except for these two reports, there have been few direct reports on the foaming properties of fungal proteins. Ishara et al. found that the FC and FS values of edible fungi were 6.1–131.5% and 3.3–69.6%, respectively, but due to the lack of protein data, the relationship of the proteins with the foam performance in edible mushrooms was not analyzed [146]. Similarly, using ultrafiltration membrane separation, Longchamp et al. obtained by-products with a good foam ability and foam stability from Quorn cell fermentation broth, but it was hard to confirm the effect of the protein because the constitutes in the by-products were too complex [147].

In accordance with the study on other functional properties, chemical reagents or physical and biological treatments can alter the foaming properties of alternative proteins. For example, during the extraction of *Tenebrio molitor* protein using alkali-soluble acid precipitation, the addition of sodium chloride and ammonium sulfate increased its content of soluble protein, α-helix, β-turn and disulfide bonds, thereby improving its foaming properties. The FC and FS values of the protein at pH 8.0 were 205% and 65.59%, respectively [128]. After being heated at 80 °C for 30 min, the major fraction globular proteins in quinoa protein isolates unfolded, and the buried hydrophobic groups were exposed, resulting in an improvement in foaming properties [148]. Similarly, Santiago et al. found that after heat treatment with sodium chloride addition, the foaming capacity of the *Locusta migratoria* protein isolate increased from 190% to 1170% and was not significantly different from that of whey protein isolate (1030%); its foam stability also increased and was not statistically different to that of whey protein isolate [111]. Enzymatic hydrolysis with proteases induced remarkable conformational changes in *Locusta migratoria* protein flour, led to the generation of small peptides and exposure of surface-stabilizing residues and resulted in an improved foam ability due to rapid diffusion and stabilization of the interfacial layer [149].

**Table 3:** Examples of the improvement in the physicochemical characteristics of alternative proteins through modification strategy.

Protein Type	Protein Resource	Modification	Improvement in Physicochemical Characteristics	Mechanism	Reference
plant protein	rapeseed protein isolates (extracted using alkali solubilization–acid precipitation)	ultrasound-assisted pH shift treatment	solubility: from 8.92% to 31.80%	The cavitation effect in the ultrasonic treatment made the hydrophobic clusters more easily exposed by disrupting strong interactions including Van de Waals forces, hydrogen bonds and dipole attractions between the molecules. Meanwhile, under the polar condition of acid/ alkaline, the protein chain was further expanded.	[100]
	pea protein isolate	acylation modification and/or guar gum conjugation	the minimum gelling concentration decreased to 7%; EC: from 45.08% to 100%; ES: from 39.66% to 100%	The protein was unfolded, and the disulfide cross-linking was enhanced during acetylation, improving the gelation properties. The unfolding of the protein was through the conjugation-enhanced protein hydrophobic interaction in the formation of a more stable gel network, reducing the amount of proteins required for gel formation. The acylated protein could form more stable layers around the oil droplets to facilitate their interaction with the aqueous phase because of the improved solubility. The conjugation of the guar gum caused the formation of a strong solvated layer at the oil–water interface, which favored the steric stabilization of the emulsion oil droplet.	[114]
	quinoa protein isolates (extracted using alkali solubilization–acid precipitation)	heat-treated at 80 ◦C for 30 min	FC: from 33.16% to 89.99% (at pH 7); FS: from 69.40% to 124.15% (at pH 7)	The major fraction globular proteins were unfolded during heat treatment, resulting in the exposure of buried hydrophobic groups, which led to an improvement in the interfacial properties of the proteins.	[148]
fungal protein	*Phlebopus portentosus* protein isolate (extracted using alkali solubilization–acid precipitation)	protein nanoparticulation using pH-cycle method	solubility: from lots of visible precipitates to no visible precipitates	A high extent of deprotonation at pH 11.5 increased the magnitude of the net negative charge of the protein molecules, leading to solubilization. When lowering the pH back to 7.0, the decrease in pH reduced the electrostatic repulsion between the protein molecules and induced molecule interaction and assembly to form nanoparticles.	[101]
algal protein	*Spirulina* protein concentrate	high-pressure homogenization	The final G’ increased by 178 ± 8% and 141 ± 5% after homogenization at 50 and 100 MPa, respectively, indicating that gel stiffness was improved, and more solid-like gels formed	High-pressure homogenization caused the dissociation of large insoluble protein aggregates to highly dispersible ones, resulting in a partial unfolding of the protein and exposure of hydrophobic amino acids. This exposure enhanced the hydrophobic interactions; thus, the gel strength increased.	[117]
	*Spirulina* phycocyanin	ultra-high-pressure composite glycation	EAI: from below 50 m^2^/g to over 120 m^2^/g	The glycation introduced hydroxyl groups into the protein, which improved its hydrophilicity. Meanwhile, ultra-high-pressure could promote the extension and exposure of hydrophobic groups on the protein to achieve a hydrophilic and lipophilic balance and improve the emulsifying ability.	[132]
	*Spirulina* phycocyanin	glutaminase deamidation and succinylation	EAI: from below 90 m^2^/g to over 150 m^2^/g (at pH 4); ESI: from below 40 min to over 60 min (at pH 4)	The exposure of the hydrophobic patches after deamidation and succinylation resulted in a better hydrophobic–hydrophilic balance that was necessary for emulsification. The improved solubility made the protein molecules more available to absorb at water–oil interfaces.	[133]
insect protein	*Locusta migratoria* L. protein flour	enzymatic hydrolysis using proteases (alcalase, neutrase, flavourzyme, papain)	FS: from below 10% to 72% at pH 7	Conformational changes induced by enzymatic cleavage led to the generation of small peptides and the exposure of surface-stabilizing residues, resulting in improved foamability due to the rapid diffusion and stabilization of the interfacial layer.	[149]

## 4. Conclusions and Perspectives

There is still a big gap between alternative proteins and animal proteins in terms of nutritional value and physicochemical activity. Though fungal proteins, algal proteins and insect proteins have shown potential to overcome some of the disadvantages of most plant proteins, such as a higher sulfur-containing amino acid content, higher digestibility and better foaming properties, their nutritional composition and technological function have still not been thoroughly explored. In future, research should be conducted on the screening of new alternative protein raw materials, the identification of their structural features and the evaluation of their edible safety, nutritional value and physicochemical properties. More importantly, considering that the nutritional quality and techno-functional properties of alternative proteins are not only dependent on their molecular properties but also affected by their extraction process and applied scenario conditions, it is crucial to remove the interference of the cell wall, pigment and carapace during protein extraction and to develop commercially available physical, chemical, biological and other novel techniques to enhance the production yield and functionality of alternative proteins. Only on this basis is it possible to combine the raw alternative protein materials in the design of formulas according to the requirement of different application scenarios to produce protein products that can satisfy consumer expectations.

## Figures and Tables

**Table 1:** Protein and essential amino content of some reported alternative protein resources.

Protein Type	Protein Resource	Protein Content (%)	Essential Amino Acid Content (%)	Reference
plant protein	defatted sesame	40.90	17.65–38.42	[20]
	soy	44.30	32.26	[21]
	green pea	24.90	38.30	[22]
	lupin	36.30	33.26	[23]
	almond	23.78–28.15	39.73–41.08	[24]
	walnut	16.65	30.08	[25]
	oat	14.34	38.38	[26]
	brown rice	9.39	39.93	[27]
	quinoa	9.15–15.46	17.9–37.45	[28]
	defatted *Moringa Oleifera* leaves	24.10	40.29	[30]
fungal protein	*Pleurotus geesteranus*	23.8–28.3	31.59–38.54	[32]
	*Fusarium venenatum*	45–54	40.80	[33]
algal protein	*Spirulina* sp.	59.16	21.80	[35]
	*Chlorella vulgaris*	53.50	39.43	[36]
insect protein	house cricket	53.90	36.95	[40]
	Bombay locust	36.31	33.23	[40]

## Data Availability

Data are contained within the article.
